# Cardiovascular manifestations identified by multi-modality imaging in patients with long COVID

**DOI:** 10.3389/fcvm.2022.968584

**Published:** 2022-09-23

**Authors:** Nobuhiro Murata, Akimasa Yamada, Hidesato Fujito, Naoki Hashimoto, Tetsuro Nagao, Yudai Tanaka, Katsunori Fukumoto, Riku Arai, Yuji Wakamatsu, Yasunari Ebuchi, Masaki Monden, Keisuke Kojima, Kentaro Hayashi, Yasuhiro Gon, Yasuo Okumura

**Affiliations:** ^1^Division of Cardiovascular Medicine, Department of Internal Medicine, Nihon University School of Medicine, Tokyo, Japan; ^2^Division of Respiratory Medicine, Department of Internal Medicine, Nihon University School of Medicine, Tokyo, Japan

**Keywords:** long COVID, CMR, SPECT, myocardial injury, pulmonary embolism

## Abstract

**Background:**

The possibility of permanent cardiovascular damage causing cardiovascular long COVID has been suggested; however, data are insufficient. This study investigated the prevalence of cardiovascular disorders, particularly in patients with cardiovascular long COVID using multi-modality imaging.

**Methods:**

A total of 584 patients admitted to the hospital due to COVID-19 between January 2020 and September 2021 were initially considered. Upon outpatient follow-up, 52 (9%) were suspected to have cardiovascular long COVID, had complaints of chest pain, dyspnea, or palpitations, and were finally enrolled in this study. This study is registered with the Japanese University Hospital Medical Information Network (UMIN 000047978).

**Results:**

Of 52 patients with long COVID who were followed up in the outpatient clinic for cardiovascular symptoms, cardiovascular disorders were present in 27% (14/52). Among them, 15% (8/52) had myocardial injury, 8% (4/52) pulmonary embolisms, and 4% (2/52) both. The incidence of a severe condition (36% [5/14] vs. 8% [3/38], *p* = 0.014) and in-hospital cardiac events (71% [10/14] vs. 24% [9/38], *p* = 0.002) was significantly higher in patients with cardiovascular disorders than in those without. A multivariate logistic regression analysis revealed that a severe condition (OR, 5.789; 95% CI 1.442–45.220; *p* = 0.017) and in-hospital cardiac events (OR, 8.079; 95% CI 1.306–25.657; *p* = 0.021) were independent risk factors of cardiovascular disorders in cardiovascular long COVID patients.

**Conclusions:**

Suspicion of cardiovascular involvement in patients with cardiovascular long COVID in this study was approximately 30%. A severe condition during hospitalization and in-hospital cardiac events were risk factors of a cardiovascular sequalae in CV long COVID patients.

## Introduction

The impact of coronavirus disease 2019 (COVID-19) on healthcare systems and economies is undoubtedly one of the worst disasters experienced by humans in recent decades. Worldwide, millions have survived COVID-19 (1), with some reporting an incomplete recovery months beyond the acute illness, a condition commonly referred to as long COVID (2). Long COVID is defined as the persistence of various symptoms beyond 4 weeks of a severe acute respiratory syndrome coronavirus 2 infection (3). Other names include post-acute COVID-19 syndrome (4), post-acute sequelae of COVID-19 (5), and long-haul COVID (6). The World Health Organization defines the post-COVID-19 condition as persistence of symptoms more than 3 months after a SARS-CoV-2 infection, lasting for at least 2 months, and not explained by any other illness (7). Long COVID is characterized by a diverse range of symptoms and signs, spanning multiple organ systems. Previous studies have revealed that over one-third of COVID-19 patients suffer from a variety of persistent symptoms, including dyspnea, chest pain, palpitations, fatigue, headache, hair loss, and an impaired sense of taste and smell, after the acute illness (8). Chest pain, palpitations, and dyspnea may be attributed to underlying cardiovascular disease, which when present with long COVID, is known as cardiovascular long COVID (CV long COVID) (9). The possibility of permanent cardiovascular damage as a cause of CV long COVID has been suggested; however, data regarding the prevalence, risk factors, associated symptoms, and prognosis remain scarce. In this study, we investigated cardiovascular involvement in patients with CV long COVID. To the best of our knowledge, this is the first study to examine the cardiovascular disorders using multi-modality imaging.

## Materials and methods

### Study population

**FIGURE 1:**
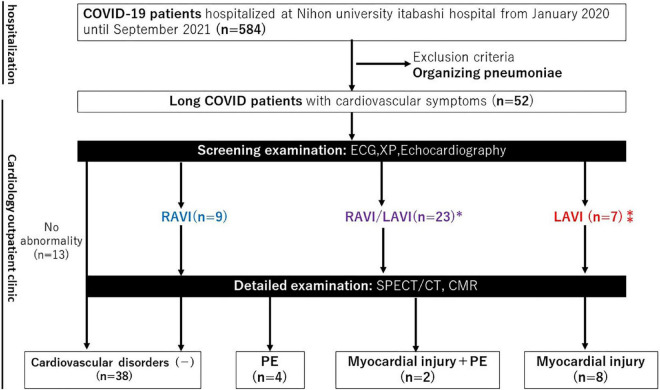
Flow chart of the study patients. CMR, cardiac magnetic resonance; ECG, electrocardiogram; LAVI, left atrial and/or ventricular involvement; PE, pulmonary embolism; RAVI, right atrial and/or ventricular involvement; SPECT/CT, single photon emission computed tomography/computed tomography; XP, X-ray photograph. *Contraindication of CMR (*n* = 4), ${}_{*}^{*}$Contraindication to CMR (*n* = 1).

We analyzed all patients admitted to Nihon University Itabashi Hospital diagnosed with COVID-19; this comprised a total of 584 patients hospitalized at our hospital from January 2020 until September 2021. During their hospital stay, we informed all 584 COVID-19 patients to visit our cardiology outpatient clinic if they had any CV long COVID symptoms (dyspnea, chest pain, and/or palpitations) after discharge. As a result, 9% (52/584) visited the cardiology outpatient clinic because of CV long COVID symptoms after discharge and were enrolled in the study (Figure 1). Patients with persistent organizing pneumonia were excluded from this study. This single center prospective observational study was approved by the Nihon University Itabashi Hospital institutional review board (IRB) (RK-210209-1) and informed consent was obtained from patients as appropriate.

### Screening protocol

At the cardiology outpatient clinic, screening tests, such as an electrocardiogram (ECG), chest X-ray, and echocardiography, were performed. The ECGs were evaluated by an experienced cardiologist according to the Sokolow-Lyon voltage criterion (10, 11). If left ventricular hypertrophy, abnormal left ventricular repolarization, non-specific ST-T changes, and left atrial enlargement were detected, the patients were defined as being suspected of having left atrial and/or ventricular involvement (LAVI). However, if right ventricular hypertrophy and right atrial enlargement were detected, the patients were defined as being suspected of having right atrial and/or ventricular involvement (RAVI). The chest X-rays were evaluated by an experienced cardiologist. If left 3rd arch and left 4th arch enlargement were detected, the patients were defined as being suspected of having LAVI. However, if right 2nd arch and left 2nd arch enlargement were detected, the patients were defined as being suspected of having right RAVI. Echocardiography was performed by an experienced sonographer and the analysis of the results was performed by two expert cardiologists (YS and TY) according to the American Society of Echocardiography guidelines (12, 13). If a low ejection fraction [<55%], wall motion abnormalities, and elevated E/e’ [>14] were detected, the patients were defined as being suspected of having LAVI. However, if a high tricuspid regurgitant pressure gradient [TRPG > 30 mmHg] and low tricuspid annular plane systolic excursion [TAPSE < 17 mm] were detected, the patients were defined as being suspected of having right RAVI. Patients suspected of having LAVI underwent cardiac magnetic resonance (CMR) imaging, those suspected of having RAVI underwent lung perfusion single-photon emission computed tomography/computed tomography (SPECT/CT), and those suspected of having LAVI/RAVI underwent both CMR and SPECT/CT (Figure 1). CMR was not performed in cases with contraindications (severe renal dysfunction with an estimated glomerular filtration rate [eGFR] of less than 30 ml/min/1.73 m^2^, pacemaker implantation, or allergy to gadolinium contrast media). CMR and SPECT/CT were chosen for the detailed examinations of this protocol because myocardial injury (14, 15) and pulmonary embolisms (PEs) (16) are common cardiovascular complications of COVID-19, according to various reports since the COVID-19 outbreak.

### Cardiac magnetic resonance and lung perfusion single-photon emission computed tomography/computed tomography

The CMR data acquisition and post processing have been described in a previous study at our hospital (17). MR images were acquired using a 1.5-T scanner (Ingenia; Philips Healthcare, Eindhoven, Netherlands) with retrospective electrocardiographic gating. The CMR protocol consisted of standard steady-state free precession cine MRI and LGE-MRI. LGE-MRI was performed with a T1-weighted inversion-recovery gradient echo sequence (slice thickness, 10 mm; slice gap, -5 mm; TR/TE, 4.6/2.2 ms; flip angle, 15°; field of view 360 mm^2^ × 360 mm^2^; acquisition matrix size, 266 × 180; reconstruction matrix size, 384 × 384; SENSE factor 2; and inversion time, 200–300 ms) 15 min after contrast agent administration (0.15 mmol/kg, Gd-BTDO3A, Gadovist; Bayer Japan, Tokyo, Japan) in three long-axis slices (two-, three-, and four-chamber) and a stack of short-axis slices completely encompassing the left ventricle. The CMR images were interpreted by experienced observers using commercial post processing software (Circle CVI 42; Circle Cardiovascular Imaging Inc., Calgary, Canada). Myocardial LGE was independently identified by 3 observers (AY, HF, and NH). Discrepancies were resolved through a consensus. The diagnosis of myocardial injury was based on confirmation of the presence of a CMR-late gadolinium enhancement (LGE) by multiple physicians in accordance with previous studies (18). The ratio of the LGE volume and the total LV myocardium volume in the LGE-positive patients was calculated using a 5 SD technique. For the pulmonary blood flow scintigraphy, after an intravenous injection of 260 MBq of ^99m^Tc-macro-aggregated albumin, SPECT imaging with low-dose CT (GCA9300A; Canon Medical Systems, Tokyo, Japan) was performed with the patient in the supine position. Planar imaging was performed in multiple projections (anterior and posterior, right and left lateral, right anterior and posterior oblique, and left anterior and posterior oblique views). The images were reconstructed in the transaxial, coronal, and sagittal views and reviewed for perfusion defects. The mismatched perfusion defects in this study were based on mismatches between the CT and scintigraphy images. The PE diagnosis was made by multiple physicians using the imaging findings of segmental or subsegmental perfusion defects in areas where no abnormalities were observed on CT images, in accordance with a previous study (19).

### Data collection

We reviewed the medical records of the patients and extracted data on the age, sex, body mass index (BMI), Clinical Frailty Scale (CFS), history of the illness, smoking, and drinking. After the initial outpatient visit, follow-up visits were ordered every 1–3 months for up to one year. Follow-up visits to assess the duration of the symptoms were conducted in the outpatient cardiology clinic of Nihon University Itabashi Hospital. All participants were interviewed face-to-face by a trained cardiologist, who assessed the presence or absence of chest pain, palpitations, and dyspnea, which have a high cardiovascular specificity among the long COVID symptoms reported in previous studies (20), using standardized questions. The in-hospital cardiac events (heart failure, arrythmia), disease severity (requiring ICU admission or ventilator management), treatment, blood tests during hospitalization, long COVID symptom clusters and duration, and blood tests after discharge were similarly recorded. A severe condition during the COVID-19 hospitalization was defined as an SpO2 of less than 93% requiring ventilator management or an ICU admission for an equivalent respiratory management according to the criteria of the Japanese Ministry of Health, Labour, and Welfare.

### Statistical analysis

Continuous variables were reported as the mean ± standard deviation (SD) or median (interquartile range [IQR]). Categorical variables were reported as the percentage and number of patients. Differences in the continuous variables between the two groups (presence/absence of cardiovascular disorders) were evaluated using the unpaired Student’s *t*-test or Mann–Whitney *U*-test. Between-group differences in the categorical variables were evaluated using the chi-square test. Comparisons of continuous and categorical variables among the three groups (population with cardiovascular disorders, myocardial injury, PE, or both) were performed using the Kruskal–Wallis test and chi-square test, respectively. A logistic regression analysis was performed to evaluate the odds ratio (OR) of the risk factors and associated long COVID symptoms. Due to the small number of cases, a multivariate analysis was performed using a propensity score based on the age, BMI, and prevalence of atrial fibrillation. A Spearman’s rank correlation was used to assess the association between the follow-up period and duration of the long COVID symptoms. All statistical analyses were performed using SPSS Statistics 24 software (IBM Corp., Armonk, NY, USA), and a *p*-value <0.05 was considered significant.

## Results

### Diagnosis

**FIGURE 2:**
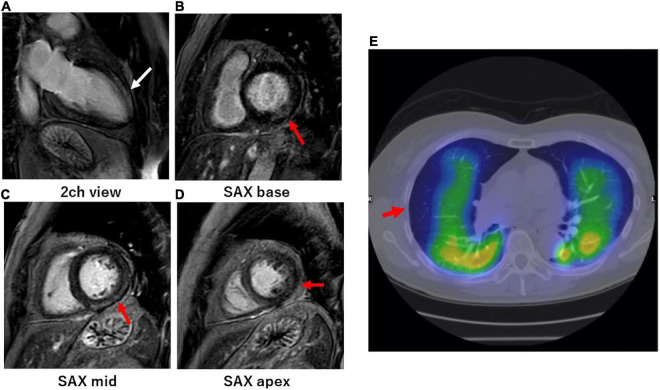
Cardiac magnetic resonance of myocardial injury and SPECT/CT of pulmonary embolisms in representative long COVID patients. **(A–D)** A woman in her 60s visited the cardiology outpatient clinic because of persistent dyspnea and palpitations. The patient was severely ill during the acute phase of a COVID-19 infection and was temporarily managed with a ventilator. She underwent CMR at the outpatient clinic 1 month after the onset of persistent dyspnea and palpitations. CMR-LGE imaging in the 2-chamber view **(A)** showed intramyocardial enhancement in the mid-distal anterior wall of the left ventricle (white arrows). CMR-LGE imaging in the short-axis views **(B–D)** also showed endomyocardial **(B)** and subepicardial **(C,D)** enhancement on the basal-distal inferolateral wall, respectively (red arrows). **(E)** A woman in her 60s visited the cardiology outpatient clinic because of persistent dyspnea and chest pain. The patient was mildly ill during the acute phase of the COVID-19 infection. Lung perfusion SPECT/CT at the outpatient clinic revealed defects in the middle and lower peripheral lesions in the right lung (red arrows). Her COVID symptoms persisted for more than 6 months.

Of the 52 patients who visited the cardiology outpatient clinic, a screening examination with an ECG, Chest X-ray, and echocardiography identified RAVI in 9, LAVI in 7, and RAVI/LAVI in 23 patients. SPECT/CT was performed in all 9 patients with RAVI and 4 patients who had RAVI/LAVI but contraindications to CMR. CMR and SPECT/CT were performed in the remaining 19 RAVI/LAVI patients; CMR was performed in 6 of the 7 patients with LAVI (1 was excluded due to a contraindication to CMR). Screening examinations identified the presence of cardiovascular disorders in 27% (14/52), i.e., 15% (8/52) had myocardial injury, 8% (4/52) PEs, and 4% (2/52) both (Figure 1). There were no specific associations between each of the screening examinations and myocardial injury and PEs, but an elevated TRPG identified PEs in 29% (2/7) and right ventricular enlargement in 50% (1/2), respectively, and ST depression identified myocardial injury in 40% (2/5), negative T waves in 40% (2/5), left 4th arch enlargement in 75% (3/4), anterior wall asynergy in 50% (1/2), left atrial P waves in 33% (1/3), and complete left bundle branch block in 50% (1/2), respectively. The site of the LGE lesion was the mid-myocardium (3/10 patients), endomyocardium (1/10 patients), and/or sub-epicardium (6/10 patients), respectively. The inferior and inferolateral wall was the most frequently involved area (8/10 patients). The median of the LGE/myocardium ratio was 7.2% (IQR, 4.2–10.9%). Images of representative cases of myocardial injury and PEs are shown in Figure 2.

### Baseline characteristics, status, and therapy

The baseline characteristics are summarized in Table 1. Patients with cardiovascular disorders tended to be older (62 ± 10 years vs. 56 ± 14 years, *p* = 0.07) and had a higher BMI (27.1 ± 6.0 vs 24.7 ± 5.4, *p* = 0.08) and higher prevalence of atrial fibrillation (AF) (21% [3/14] vs. 5% [2/38], *p* = 0.08). There were no differences in the sex, CFS, smoking and drinking history, prevalence of diabetes mellitus, dyslipidemia, hypertension, hyperuricemia, chronic kidney disease, history of coronary artery disease, heart failure, strokes, chronic obstructive lung disease/asthma, and malignant tumors between the two groups. The status and therapy profiles are shown in Table 1. The incidence of a severe condition (36% [5/14] vs. 8% [3/38], *p* = 0.014), and in-hospital cardiac events (71% [10/14] vs. 24% [9/38], *p* = 0.002) was significantly higher in patients with cardiovascular disorders. There was no significant difference between the two groups regarding drug treatment, such as with steroids, antiviral drugs, antibody cocktails, antirheumatic drugs, and anticoagulants. In terms of the peak biomarker values during the hospitalization, there were no differences in the other biomarkers, such as the troponin I, N-terminal pro-brain natriuretic peptide (NTproBNP), and D-dimer. In patients with cardiovascular disorders, when qualitatively comparing the subgroups with myocardial injury, PEs, and both, the patients with PEs were more frequently female. The incidence of using oxygen therapy, in-hospital cardiac events, and the use of anti-rheumatic drugs were higher in the patients with myocardial injury or both myocardial injury and PEs than in those with a PE alone.

**TABLE 1:** Clinical characteristics of patients with and without cardiovascular disorders.

	CVD (-) (*n* = 38)	CVD (+) (*n* = 14)	*P*-value*	Mi^†^ (*n* = 8)	PE^†^ (*n* = 4)	Mi + PE^†^ (*n* = 2)
Age, year	56 ± 14	62 ± 10	0.07	64 ± 10	66 ± 4	49 ± 13
Male, *n* (%)	27 (71)	10 (72)	0.98	7 (88)	1 (25)	2 (100)
BMI, kg/m^2^	24.7 ± 5.4	27.1 ± 6.0	0.08	26.7 ± 5.4	26.0 ± 8.2	31.0 ± 4.6
Clinical Frailty Scale	2.3 ± 0.8	2.3 ± 0.8	0.41	2.4 ± 1.1	2.3 ± 0.5	2.0 ± 0
Smoking, *n* (%)	18 (48)	7 (50)	0.87	3 (38)	2 (50)	2 (100)
Alcohol, *n* (%)	21 (55)	9 (64)	0.56	6 (67)	2 (50)	1 (50)
DM, *n* (%)	6 (16)	4 (29)	0.30	2 (25)	1 (25)	1 (25)
DLP, *n* (%)	12 (32)	2 (14)	0.21	1 (13)	0 (0)	1 (50)
HTN, *n* (%)	20 (53)	7 (50)	0.87	5 (63)	2 (50)	0 (0)
HUA, *n* (%)	4 (11)	4 (29)	0.11	3 (38)	1 (25)	0 (0)
CKD, *n* (%)	9 (24)	2 (14)	0.46	1 (13)	0 (0)	1 (50)
AF, *n* (%)	2 (5)	3 (21)	0.08	2 (25)	1 (25)	0 (0)
History of CAD, *n* (%)	9 (24)	2 (14)	0.46	1 (13)	1 (25)	0 (0)
History of HF, *n* (%)	6 (16)	0 (0)	0.11	0 (0)	0 (0)	0 (0)
History of a Stroke, *n* (%)	1 (3)	1 (7)	0.45	1 (13)	0 (0)	0 (0)
COPD/Asthma, *n* (%)	3 (8)	0 (0)	0.28	0 (0)	0 (0)	0 (0)
Malignant tumor, *n* (%)	1 (3)	0 (0)	0.54	0 (0)	0 (0)	0 (0)
**Hospitalization**						
Severe condition, *n* (%)	3 (8)	5 (36)	0.014	4 (50)	0 (0)	1 (50)
Oxygen therapy, *n* (%)	20 (71)	10 (71)	0.22	7 (88)	1 (25)	2 (100)
Ventilator, *n* (%)	0 (0)	3 (21)	0.003	3 (38)	0 (0)	0 (0)
ECMO, *n* (%)	0 (0)	1 (7)	0.09	1 (13)	0 (0)	0 (0)
Cardiac event, *n* (%)	9 (24)	10 (71)	0.002	7 (88)	1 (25)	2 (100)
Steroid, *n* (%)	31 (82)	13 (93)	0.32	8 (100)	3 (75)	2 (100)
Antiviral drug, *n* (%)	30 (79)	12 (86)	0.58	8 (100)	2 (50)	2 (100)
Antibody cocktail, *n* (%)	3 (8)	1 (7)	0.93	0 (0)	1 (25)	0 (0)
Anti-rheumatic drug, *n* (%)	11 (29)	7 (50)	0.16	5 (63)	0 (0)	2 (100)
Anticoagulant, *n* (%)	21 (55)	10 (71)	0.29	7 (88)	2 (50)	1 (50)
Peak Troponin I, ng/ml (No. of patients with an abnormal value of >0.01 ng/mL)	(0.01, 0.02) 11 (29)	0.01 (0.01, 0.29) 6 (43)	0.11 0.34	0.04 (0.01, 6.94) 5 (63)	0.01 (0.01, 0.01) 0 (0)	0.02 (0.02) 1 (50)
Peak NTproBNP, pg/ml (No. of patients with an abnormal value of > 125 pg/mL)	67 (26, 285) 13 (34)	164 (27, 3,375) 7 (50)	0.44 0.30	1,870 (105, 5,090) 6 (75)	41 (18, 239) 1 (25)	60 (10) 0 (0)
Peak D-dimer, μg/ml (No. of patients with an abnormal value of > 1.0μg/ml)	1.5 (1.0, 4.3) 22 (58)	3.0 (1.0, 9.1) 10 (71)	0.15 0.37	6.0 (1.5, 11.9) 7 (88)	1.8 (1.0, 3.3) 2 (50)	4.7 (1.0) 1 (50)
**Outpatient clinic**						
Dyspnea, *n* (%)	33 (87)	13 (93)	0.55	7 (88)	4 (100)	2 (100)
Chest pain, *n* (%)	10 (26)	5 (36)	0.51	1 (13)	4 (100)	0 (0)
Palpitation, *n* (%)	5 (13)	5 (36)	0.07	3 (38)	2 (50)	0 (0)
Other symptoms, *n* (%)	12 (32)	4 (29)	0.84	2 (25)	2 (50)	0 (0)
Symptom duration, week	6 (4, 12)	11 (4, 20)	0.09	11 (4, 12)	20 (11, 41)	4 (4, 4)
Anticoagulant, *n* (%)	10 (26)	6 (43)	0.25	4 (50)	1 (25)	1 (50)
Troponin I, ng/ml (No. of patients with an abnormal value of >0.01 ng/mL)	(0.01, 0.01) 6 (16)	(0.01, 0.01) 2 (14)	0.69 0.89	(0.01, 0.25) 2 (25)	(0.01, 0.01) 0 (0)	(0.01, 0.01) 0 (0)
NTproBNP, pg/ml (No. of patients with an abnormal value of >125 pg/mL)	46 (30, 256) 12 (32)	122 (51, 280) 7 (50)	0.68 0.22	167 (70, 697) 5 (63)	58 (48, 173) 1 (25)	103 (10) 1 (25)
D-dimer, μg/ml (No. of patients with an abnormal value of > 1.0μg/ml)	1.0 (1.0, 1.03) 9 (24)	1.0 (1.0, 1.0) 2 (14)	0.64 0.46	1.0 (1.0, 1.0) 1 (13)	1.0 (1.0, 1.0) 0 (0)	1.2 (1.0) 1 (50)

Values are shown as the mean ± SD, median (interquartile range), or n (%). AF, atrial fibrillation; BMI, body mass index; CAD; coronary artery disease; CKD, chronic kidney disease; COPD, chronic obstructive pulmonary disease; CVD, cardiovascular disorder; DM, diabetes mellitus; DLP, dyslipidemia; ECMO, Extracorporeal Membrane Oxygenation; HF, heart failure; HTN, hypertension; HUA, hyperuricemia; Mi, myocardial injury; NTproBNP, N-terminal pro-brain natriuretic peptide. PE, pulmonary embolism. Severe condition was a condition requiring ICU admission or ventilator management.

*P-value by the Student’s t-test/Mann–Whitney U test or chi-squared test, as appropriate.

^†^The analyses were exploratory so a multiple correction was not applied.

### Factors associated with cardiovascular disorders

Univariate and multivariate logistic regression analyses evaluated the factors associated with cardiovascular disorders (Table 2). A severe condition (OR, 5.789; 95% CI 1.442–45.220; *p* = 0.017) and in-hospital cardiac events (OR, 8.079; 95% CI 1.306–25.657; *p* = 0.021) were independent risk factors of cardiovascular disorders in long COVID patients.

**TABLE 2:** Logistic regression analysis of cardiovascular disorders in CV long COVID patients.

	Crude OR (95% CI)	*P*-value	Adjusted OR (95% CI)	*P-value
Age	1.0 (0.9–1.1)	0.14		
BMI	1.1 (0.9–1.2)	0.17		
AF	5.0 (0.7–33.2)	0.10		
Severe condition during hospitalization	6.5 (1.3–32.4)	0.023	5.8 (1.3–25.7)	0.021
In hospital cardiac event	8.1 (2.0–32.0)	0.003	8.1 (1.4–45.2)	0.017
Palpitation	3.7 (0.9–15.5)	0.08		
Symptom duration	1.1 (0.9–1.1)	0.09		

In-hospital cardiac events were heart failure and arrhythmias. A severe condition was a condition requiring an ICU admission or ventilator requirement. AF, atrial fibrillation; BMI, body mass index; OR, odds ratio; 95% CI, 95% confidence interval.

*Adjusted by propensity scores by the age, BMI, and prevalence of atrial fibrillation.

### Long COVID symptoms and clinical outcomes

**FIGURE 3:**
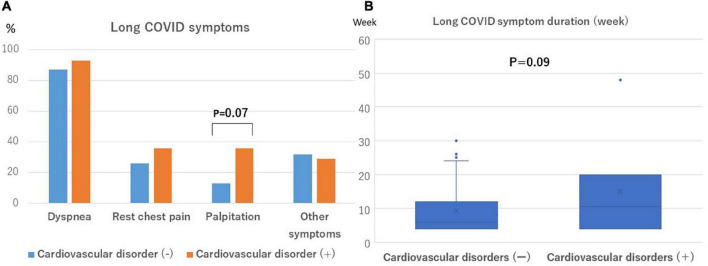
Frequency and duration of long COVID symptoms. **(A)** Palpitations are more likely to be observed in patients with cardiovascular disorders (*P* = 0.07). **(B)** The symptom duration was more likely to be longer in patients with cardiovascular disorders (*P* = 0.09).

The time from the onset to hospitalization in this cohort of patients was a median of 5 days (IQR, 3–7 days), the length of the hospitalization was a median of 13 days (IQR, 10–24 days), and the time from discharge to the initial outpatient visit was a median of 21 days (IQR, 15–29 days), respectively. The aforementioned total time from the onset to the initial outpatient visit for long COVID symptoms was a median of 40 days (IQR, 35–52 days). The long COVID symptoms are shown in Table 1. Palpitations tended to be more frequent (36% [5/14] vs. 13% [5/38], *p* = 0.07), and the duration of the long COVID symptoms from the onset of the illness tended to be longer (11 weeks [IQR, 4–20weeks] vs. 6 weeks [IQR, 4–12 weeks], *p* = 0.09) in the group with cardiovascular disorders (Figure 3). A closer look at the myocardial injury and PEs revealed that PE patients more often presented with chest pain. The median follow-up period was 163 days (IQR, 60–300 days). There was no correlation between the follow-up period and duration of long COVID symptoms (*r* = 0.33, *p* = 0.07). No deaths or hospitalizations due to cardiovascular events occurred during the observation period.

## Discussion

In this study, we investigated the cardiovascular manifestations of CV long COVID patients by multi-modality imaging methods. The major findings of our study were as follows: (1) 9% of COVID-19 patients presented to the cardiology outpatient clinic after discharge for CV long COVID symptoms; (2) 27% of CV long COVID patients had some cardiovascular disorders. Of those, 15% had myocardial injury, 8% had PE, and 4% had both complications; (3) an in-hospital severe condition and cardiac events were independent risk factors of a cardiovascular disorder in CV long COVID patients; and (4) during the observation period, CV long COVID patients who did or did not have cardiovascular disorders had no major adverse events.

### Prevalence of cardiovascular long COVID

In our registry, approximately 10% of the patients had CV long COVID symptoms (dyspnea, palpitations, and/or chest pain) and visited the cardiology outpatient clinic after discharge. In a meta-analysis regarding long COVID, the median (IQR) proportion of COVID-19 survivors experiencing at least one long COVID episode at 1 month was 54.0% (45.0–69.0%; 13 studies), at 2–5 months, 55.0% (34.8–65.5%; 38 studies), and at 6 or more months, 54.0% (31.0–67.0%; 9 studies) (21). Dyspnea is a non-specific symptom; however, chest pain and palpitations are common cardiovascular manifestations of long COVID in survivors of COVID-19 (21). The median (IQR) frequency of chest pain and palpitations is 13.3% (8.8–17.8%; 14 studies) and 9.3% (6.0–10.8%; 5 studies), respectively (21), which was similar to the proportion of patients presenting to the cardiology outpatient clinic with CV long COVID symptoms in this study. Although there are few previous reports of outpatient consultation and treatment specifically for cardiovascular symptoms of long COVID, approximately 10% of our patients presented with cardiovascular symptoms, suggesting that cardiologists should participate in the management of long COVID.

### Cardiovascular disorders in cardiovascular long COVID patients

In CV long COVID patients, 15% had a myocardial injury. Several studies on echocardiography abnormalities have been conducted since the beginning of the COVID-19 epidemic (22, 23). While, Puntmann et al. reported that CMR revealed cardiac involvement in 78% of patients and ongoing myocardial inflammation in 60% of patients in post-COVID-19 German patients (14). Brito et al. reported that approximately 30% of previously healthy college athletes in the U.S. recovering from COVID-19 showed imaging features of a resolving pericardial inflammation (15). The frequency of myocardial injury caused by COVID-19 varied in previous studies and was relatively lower in this study. This may be because the patients in this study were mainly those who presented to the cardiology outpatient clinic with CV long COVID symptoms and they were diagnosed by our specific screening examination. A study by Clark et al. with a similar patient population reported that among soldiers with cardiopulmonary symptoms in the late convalescent phase of recovery from SARS-CoV-2, only 8% of the patients had a myocardial injury as detected by CMR (9). Several mechanisms have been proposed to account for myocardial injury, including direct cytotoxic injury (1), dysregulation of the renin-angiotensin-aldosterone system (24), endotheliitis and thromboinflammation (25), and a dysregulated immune response with cytokine release (26). In addition, findings of ST depression, negative T waves, left 4th arch enlargement, asynergy, left atrial P waves, and complete left bundle branch block in the screening tests in this study may be detected in patients with myocardial injury. When these findings are detected in an outpatient setting, CMR and other techniques may be used to make an appropriate diagnosis of myocardial injury. Of the patients with CV long COVID, 8% were diagnosed with peripheral PEs based on the lung perfusion SPECT/CT findings. There have been various reports on the association between COVID-19, thromboses, and abnormal coagulation fibrinolysis since the early stages of the epidemic. It is widely known that an elevated blood D-dimer is often observed, especially in the acute phase of the disease (27). An elevated D-dimer level during a COVID-19 hospitalization is an independent risk factor for an acute thrombosis and death (28, 29). The factors that cause thrombi in various organs during COVID-19 are thought to be an infection of the vascular endothelial cells expressing ACE2 by SARS-CoV2, subsequent damage to vascular endothelial cells, and cytokine storms (30). Some case reports have mentioned PEs as a symptom of long COVID (31); however, most long COVID cases have large PEs that can be detected by contrast-enhanced CT, and there have been few reports involving peripheral PEs and long COVID that can only be detected by lung perfusion SPECT/CT. Among the screening tests in this study, findings such as an elevated TRPG and right ventricular enlargement were characteristic of patients diagnosed with PEs. If these findings are detected in an outpatient setting, SPECT or other procedures may be useful in making an appropriate diagnosis of the PE. In the present study, an in-hospital severe condition and cardiac events were independent risk factors of cardiovascular disorders in CV long COVID patients. Although not statistically significant, patients with cardiovascular disorders tended to be older and had a higher BMI, which are risk factors for the COVID-19 severity, and they also tended to have more palpitations and a longer duration of symptoms. Among the patients with cardiovascular-specific symptoms, a history of severe or cardiac events during hospitalization and a long duration of palpitations and symptoms suggested a high probability of the presence of a cardiovascular disorder and may require special attention in this patient group.

### Prognosis of cardiovascular long COVID patients

During a median follow-up of 163 days (IQR, 60–300 days), no deaths or hospitalizations due to cardiovascular events were observed among the patients. The duration of symptoms was 11 weeks (IQR, 4–20 weeks) in patients with cardiovascular disorders and 6 weeks (IQR, 4–12 weeks) in those without cardiovascular disorders. In this study, even those who visited the cardiology outpatient clinic with CV long COVID symptoms improved within approximately 12 weeks on average, and no major adverse events occurred during the observation period. Weber et al. reported that among the COVID-19 patients who survived their index hospitalization, the incremental mortality through 1 year was low, even among troponin-positive patients (32). However, few previous studies have reported the prognoses for groups of patients with long COVID symptoms. This may be because long COVID itself is unlikely to have organic cardiovascular disease, and even if it does, it is a very mild condition. However, patients with long COVID with cardiovascular complications tended to have longer-lasting symptoms of long COVID, however, the prognosis was not worse. Early detection of cardiovascular problems in cardiology for symptomatic long COVID patients may inform patients of the duration of symptoms and allow symptoms to be shortened through appropriate therapeutic intervention.

### Limitations

This study had several limitations. First, as it was conducted in a prospective observational fashion, causal relationships could not be established. Because of the selection bias that existed in this cohort due to the presence of those who did not accept the physician’s suggestion of an outpatient visit, the small proportion of women and patients in this study with a history of heart failure and coronary artery disease with little or no left ventricular systolic dysfunction, unmeasured confounders should be considered when interpreting the results. In particular, our results were based on the data obtained through specific screening examinations, which have a different sensitivity and specificity in identifying a cardiac pathology at the cardiology outpatient clinic; therefore, because symptomatic potential pulmonary disease and asymptomatic potential cardiac disease might be missed, our results regarding the use of imaging modalities and clinical outcomes between facilities should be considered descriptive. Second, due to the low number of clinical events, this study included a small number of patients, and we could not perform a statistical analysis of the factors associated with myocardial injury and PEs. In addition, the number of clinical events may not have been large enough to obtain statistical significance. Third, in the CMR analysis of this study, mainly a qualitative evaluation of the LGE was performed, but T1mapping, T2mapping, and a strain analysis were not performed. The purpose of this study was to clarify the incidence of overt myocardial injury in patients with long COVID. Therefore, subtle left ventricular and right ventricular dysfunction might have been missed. Finally, the clinical data before the COVID-19 hospitalization was lacking; hence, whether cardiovascular disorders existed before the hospitalization could not be determined.

### Conclusion

The suspicion of cardiovascular involvement in CV long COVID patients was approximately 30%. A severe condition during hospitalization and in-hospital cardiac events were risk factors of a cardiovascular sequelae in CV long COVID patients.

## Data availability statement

The original contributions presented in this study are included in the article/supplementary material, further inquiries can be directed to the corresponding author.

## Ethics statement

The studies involving human participants were reviewed and approved by Institutional Review Board (IRB) of Nihon University Itabashi Hospital (RK-210209-1). The patients/participants provided their written informed consent to participate in this study. Written informed consent was obtained from the individual(s) for the publication of any potentially identifiable images or data included in this article.

## Author contributions

NM, KH, YG, and YO contributed to the conception and design of the study. NH, TN, YT, KF, RA, YW, YE, MM, and KK organized the database. NM performed the statistical analysis and wrote the first draft of the manuscript. HF and AY wrote sections of the manuscript. All authors contributed to the manuscript revision, read, and approved the submitted version.
